# Death Certificates

**Published:** 1896-02-22

**Authors:** 


### Editor's Letter-Box.

[Our correspondents are reminded that prolixity is a great bar to publication, and that brevity of style and conciseness of statement greatly facilitate early insertion.]

### DEATH CERTIFICATES.

Dr. Augustus Bampton writes: Will you allow me to call attention to a paragraph in the leaderette on "Coroners and Practitioners," where certain instructions are given as to the correct filling up of the "primary" and "secondary" cause of death in death certificates? It is very important that all practitioners should mean the same thing when they give the "primary" cause of death. In my experience most practitioners mean by the primary cause of death, the ultimate, final, and actual mode of ending of the disease. By the examples given you appear to infer that the primary cause of death and the primary cause of the disease are the same thing, or synonymous terms. I should fill up the certificate thus—1. Primary cause of death, syncope. 2. Primary cause, peritonitis. 3. Primary cause, illegal operation—where the illegal operation is the primary cause of the disease, and really the exciting cause of death, but not the mode of dying. The primary disease is not asked to be given under No. 1 heading, although many practitioners fill up their certificates in that belief, and thus confound the statisticians.

[We think our correspondent cannot have read the model form sent out with every book of certificates by the Registrar-General, in which, as an example of how the entries are to be made, a case is given thus: Primary; Typhus, 19 days. Secondary; Pneumonia, 3 days.—Ed. *T. H.*]

